# A Fluid Dynamic In Vitro System to Study the Effect of Hyaluronic Acid Administration on Collagen Organization in Human Skin Explants

**DOI:** 10.3390/ijms26115397

**Published:** 2025-06-04

**Authors:** Andrea Galvan, Maria Assunta Lacavalla, Federico Boschi, Barbara Cisterna, Edoardo Dalla Pozza, Enrico Vigato, Flavia Carton, Manuela Malatesta, Laura Calderan

**Affiliations:** 1 Department of Neurosciences, Biomedicine and Movement Sciences, University of Verona, 37134 Verona, Italy; andrea.galvan@univr.it (A.G.); mariaassunta.lacavalla@univr.it (M.A.L.); barbara.cisterna@univr.it (B.C.); manuela.malatesta@univr.it (M.M.); laura.calderan@univr.it (L.C.); 2 Department of Diagnostic and Public Health, University of Verona, 37134 Verona, Italy; 3 Department of Engineering for Innovation Medicine, University of Verona, 37100 Verona, Italy; federico.boschi@univr.it; 4 Department of Plastic and Reconstructive Surgery, Verona University Hospital (A.O.U.I. Verona), 37126 Verona, Italy; edoardo.dallapozza@aovr.veneto.it (E.D.P.); enrico.vigato@aovr.veneto.it (E.V.); 5 Department of Cellular, Computational and Integrative Biology (CIBIO), University of Trento, 38123 Trento, Italy; 6 Center for Medical Sciences (CISMed), University of Trento, 38122 Trento, Italy

**Keywords:** 3D technology, skin, hyaluronic acid, morphology, light microscopy, electron microscopy

## Abstract

Hyaluronic acid (HA) is an unbranched polysaccharide particularly abundant in the extracellular matrix (ECM) of soft connective tissues. In humans, about 50% of the total HA in the organism is localized in the skin. HA plays an essential role in the hydration of the ECM, in the regulation of tissue homeostasis, in the resistance to mechanical stimuli/forces, and in the modulation of tissue regeneration. For these reasons, HA is widely used in regenerative medicine and cosmetics. In this study we used an innovative fluid dynamic system to investigate the effects of a cross-linked macrostructural HA formulation on dermal collagen of healthy human skin explants. The good preservation of skin explants provided by the bioreactor allowed applying refined high-resolution microscopy techniques to analyze in situ the HA-induced modifications on the ECM collagen fibrils up to 48 h from the application on the skin surface. Results demonstrated that this HA formulation, commercially proposed for subcutaneous injection, may act on dermal ECM also when applied transcutaneously, improving ECM hydration and modifying the organization of the collagen fibrils. These findings, obtained by the original combination of explanted human skin use with an advanced culture system and multiscale imaging techniques, are consistent with the volumizing and anti-aging effect of HA.

## 1. Introduction

Hyaluronic acid (HA) is an unbranched polysaccharide made of repeating disaccharides of D-glucuronic and N-acetyl-D-glucosamine linked via glycoside bonds [1,2]. It is present in almost any organ in vertebrates, and it is particularly abundant in the extracellular matrix (ECM) of soft connective tissues. In humans, HA is especially present in eyes, joints, and skin, where about 50% of the total HA in the organism is localized [3]. In detail, in the skin, HA builds up a high fraction of the ECM of the dermis, but it is also present in the epidermis, in the matrix surrounding keratinocytes and corneocytes [4,5]. In the skin, HA is mainly produced by fibroblasts and keratinocytes [3,4], while its degradation is dependent on the enzymatic activity of the fibroblasts’ hyaluronidases [6].

HA is a highly water-soluble molecule thanks to the presence of carboxyl groups and can trap a huge amount of water, about 1000 times its own weight, forming a highly viscous solution with unique viscoelastic properties [1,7]. Thanks to these peculiar chemical-physical properties, HA plays an essential role in the hydration of the ECM, in the regulation of tissue homeostasis, and in the resistance to mechanical stimuli/forces [7]. Moreover, HA is considered one of the key players in tissue regeneration; in fact, via specific receptors, HA modulates cell proliferation, differentiation, and migration, as well as inflammation and angiogenesis, which are the main processes in wound healing [8,9]. For these reasons, HA is used in medicine for tissue repair and diverse regenerative purposes [6,10,11], and in cosmetics, for its protective, moisturizing and anti-aging effects on the skin [2,7,12]. In particular, high molecular weight HA has an anti-inflammatory effect and supports tissue integrity [1,7].

In the ECM, HA interacts with other matrix proteins, in particular with the collagen fibrils, forming complex supermolecular structures that maintain skin hydration and elasticity and support mechanical integrity, stiffness, and resistance to shear forces [13]. Moreover, binding a great number of water molecules, it is noteworthy the ability of HA to form an occlusive layer on the skin surface. Indeed, the high-molecular-weight HA has been demonstrated to prevent transepidermal water loss in the epidermis, shielding it from harmful external factors and supporting optimal hydration levels [14]. In the skin, collagen is the major structural protein that forms a fibrous network mostly located in the dermis, where its framework, strength, and firmness are crucial for preventing sagging and for maintaining the skin shape. Moreover, collagen contributes to skin elasticity and hydration, playing a vital role in retaining moisture [15,16]. Collagen is differently organized in the upper dermis layer (the papillary dermis) and in the deeper layer (the reticular dermis): the papillary dermis contains thin and randomly oriented collagen fibrils, forming a delicate network that provides a nurturing and flexible interface with the epidermis; on the contrary, the reticular dermis is characterized by thicker, larger, and more organized collagen bundles, which are often parallel arranged. This layer provides the skin with its primary structural support and tensile strength [17].

In this study, we aimed at investigating in situ the effect of transdermal administration of HA on the molecular morphology and organization of the dermal collagen in human skin. The scientific approach of this study lies in its innovative combination of methodologies. Specifically, the use of human skin explants provides a model system able to retain the phenotypic characteristics of the original tissue, maintaining its complex three-dimensional (3D) cytoarchitecture, including appendages (e.g., hair, sweat, and sebaceous glands), the native ECM niche, and the original spatial relationship between cells. The ex vivo preservation is enhanced over time by using a dynamic culture system able to improve the structural and functional preservation of the skin biopsies, as it increases the oxygen/nutrient supply while promoting faster catabolite removal. [18,19]. Finally, the application of multiscale imaging techniques provides a more comprehensive understanding of complex biological systems, offering the possibility to analyze events occurring across a wide range of organization levels. By synergistically integrating the use of explanted healthy human skin, a bioreactor-based system, and multiscale imaging techniques, this work offers a comprehensive insight into the effect of HA administration on collagen organization in human skin explants.

## 2. Results

### 2.1. Light Microscopy (LM)

**Figure 1:**
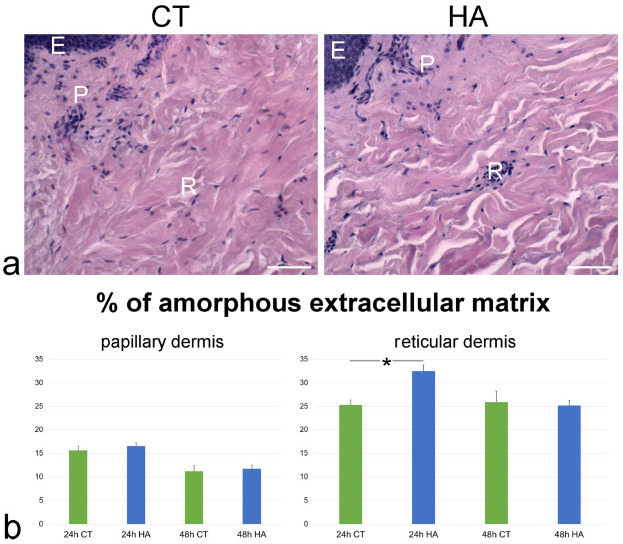
(**a**) LM images of dermis in control (CT) and HA-treated skin samples (HA) at 24 h. Hematoxylin and eosin staining. E, epithelium; P, papillary dermis; R, reticular dermis. Bars, 50 µm. (**b**) Mean ± SE values (*n* = 40) of the percentage of amorphous extracellular matrix in papillary and reticular dermis of control (CT) and hyaluronic acid-treated (HA) skin samples at 24 and 48 h. The asterisk (*) indicates a statistically significant difference between HA-treated samples and the relative CT.

At bright-field microscopy, both control and HA-treated healthy skin samples showed the typical histological architecture composed of an epithelial outer layer (the epidermis, made of closely packed cells) and a connective inner layer (the dermis, characterized by abundant ECM and scattered cells) (Figure 1). In the dermis, two layers were recognizable: the upper papillary dermis was dense and compact, while the deeper reticular dermis appeared less dense due to the occurrence of amorphous spaces among the fibrillar component. At 24 h, in HA-treated skin the reticular dermis appeared looser than in control samples (Figure 1a), while no difference was evident after 48 h.

The quantitative evaluation of the amorphous ECM (i.e., the spaces among the fibrillar component), expressed as its percentage of the total dermal connective tissue, confirmed the more compact arrangement of papillary dermis, in comparison to reticular dermis. In fact, in control samples the percentage of amorphous ECM ranged from about 11% to 16% in the papillary dermis while it ranged from 23% to 28% in the reticular dermis (Figure 1b). In control samples, the amorphous component of papillary dermis was significantly more abundant at 24 h than at 48 h (*p* = 0.01), whereas in reticular dermis no significant difference was found between 24 and 48 h (*p* = 0.07).

As for the effect of HA treatment, at 24 h a statistically significant increase (*p* < 0.001) of the amorphous component in the reticular dermis was found in HA-treated skin vs. control (Figure 1b), thus confirming the histological observation. Conversely, no statistically significant difference was found between control and HA-treated samples in the papillary dermis at 24 h (*p* = 0.96) and 48 h (*p* = 0.91) or in the reticular dermis at 48 h (*p* = 0.39).

### 2.2. Scanning Electron Microscopy (SEM)

**Figure 2:**
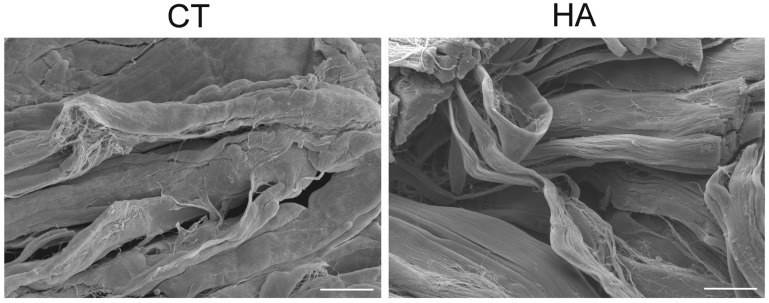
SEM images of reticular dermis in control (CT) and hyaluronic acid-treated (HA) skin at 24 h. Note the looser arrangement and the tortuous pattern of collagen in HA. Bars, 20 µm.

Observations at SEM of the dermal layers showed many wavy bundles of collagen fibrils oriented in different directions; in the papillary dermis the bundles were closely arranged, while in the reticular dermis they were less compact. No morphological difference was observed in papillary dermis at both 24 and 48 h. In the reticular dermis of HA-treated skin, at 24 h collagen bundles showed a tortuous pattern and a loose arrangement, thus delimiting large free spaces, while in control samples the bundles were more linear and closer to each other (Figure 2). No difference was found at 48 h between control and treated skin.

### 2.3. Transmission Electron Microscopy (TEM)

**Figure 3:**
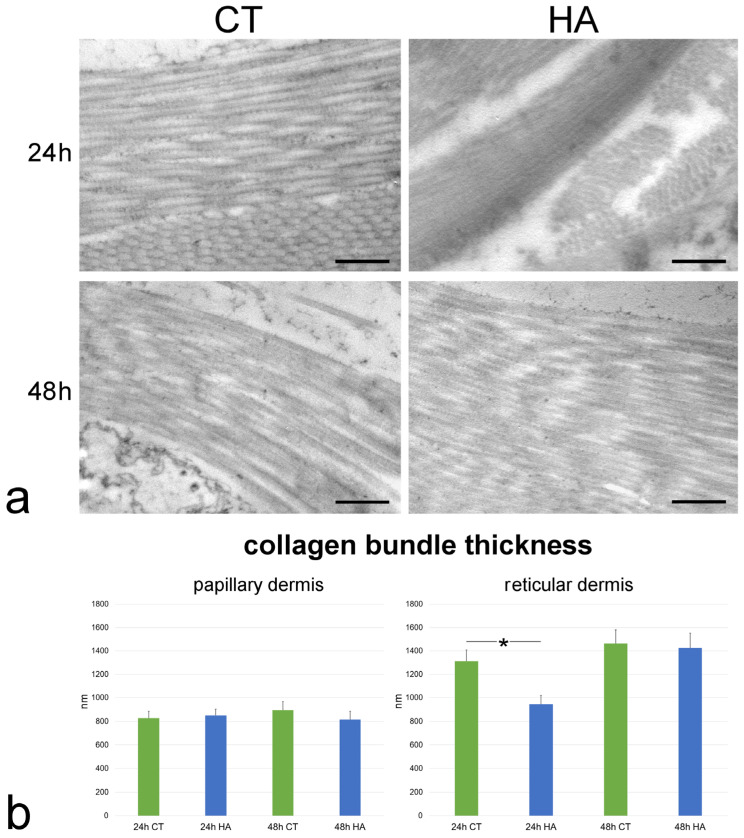
(**a**) TEM images showing collagen bundles in the reticular dermis of control (CT) and hyaluronic acid (HA)-treated skin. Bars, 500 nm. (**b**) Mean ± SE values (*n* = 40) of collagen bundle thickness in papillary and reticular dermis of control (CT) and hyaluronic acid-treated (HA) skin samples at 24 and 48 h. The asterisk (*) indicates a statistically significant difference between HA-treated samples and the relative CT.

No ultrastructural difference in the collagen bundles of papillary and reticular dermis was observed at TEM between control and HA-treated skin. In fact, collagen bundles were always oriented in different directions, and each bundle was composed of parallel- arranged collagen fibrils, devoid of branches or anastomoses (Figure 3a).

Morphometric evaluation performed at TEM revealed that collagen bundle thickness of control samples was similar at 24 and 48 h in both papillary and reticular dermis (*p* = 0.72 and *p* = 0.34, respectively). Moreover, the collagen bundle thickness was higher in the reticular vs. papillary dermis at both 24 and 48 h (*p* < 0.001 for both comparisons).

Following HA treatment, collagen bundle thickness underwent a significant decrease in the reticular dermis at 24 h (*p* = 0.04) (Figure 3b). On the contrary, no significant difference was found between control and HA-treated samples in the papillary dermis at 24 h (*p* = 0.69) and 48 h (*p* = 0.43) or in the reticular dermis at 48 h (*p* = 0.83).

**Figure 4:**
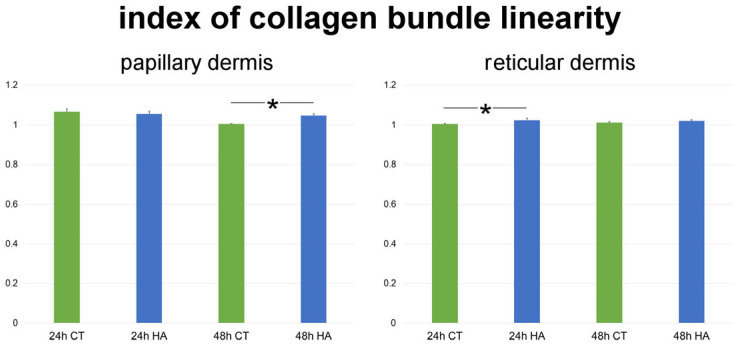
Mean ± SE values (*n* = 40) of the index of collagen bundle linearity in papillary and reticular dermis of control (CT) and hyaluronic acid-treated (HA) skin samples at 24 and 48 h. Asterisks (*) indicate statistically significant differences between HA-treated samples and the relative CT.

The index of collagen bundle linearity refers to the ratio between the real length of the bundle profile and the corresponding linear length; therefore, the higher the index value, the higher the bundle tortuosity. In control samples, the index significantly decreased at 48 h in comparison to 24 h in papillary dermis (*p* < 0.001), while it did not change in reticular dermis (*p* = 0.87). Skin samples exposed to HA treatment showed significantly higher values of collagen bundle linearity after 48 h in papillary dermis (*p* < 0.001) as well as after 24 h in reticular dermis (*p* = 0.003). Conversely, no statistically significant difference was found between HA-treated and control samples after 24 h in papillary dermis (*p* = 0.31) and after 48 h in reticular dermis (*p* = 0.47) (Figure 4). Values of HA-treated samples were similar at 24 and 48 h in both papillary and reticular dermis (*p* = 0.40 and *p* = 0.52, respectively).

The size of single collagen fibrils was found to be significantly smaller at 24 h vs. 48 h in both the papillary and reticular dermis of control samples (both *p* < 0.001). The values were statistically similar between the papillary and reticular dermis at both 24 h (*p* = 0.44) and 48 h (*p* = 0.19).

**Figure 5:**
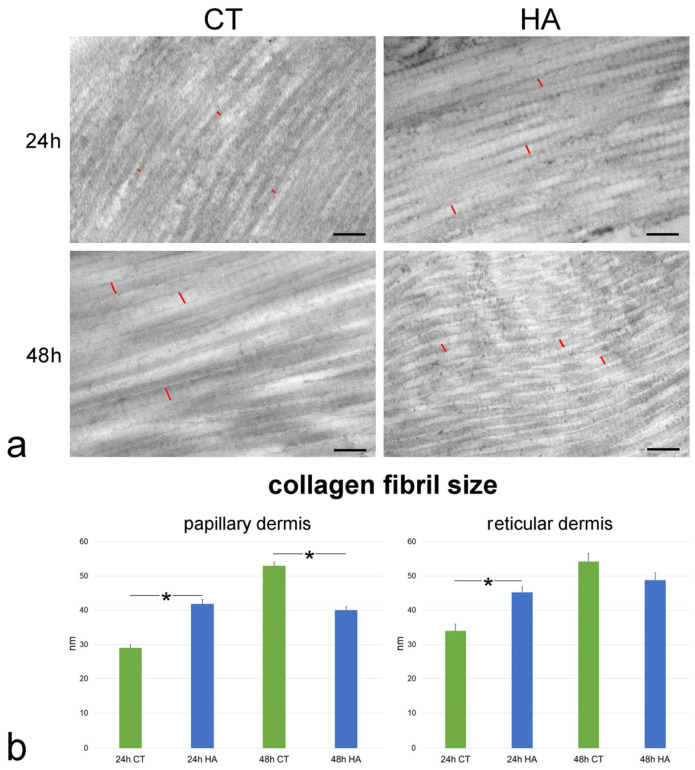
(**a**) TEM images of collagen fibrils in the papillary dermis of control (CT) and hyaluronic acid (HA)-treated skin. Red lines indicate the size of some collagen fibrils. Bars, 200 nm. (**b**) Mean ± SE values (*n* = 40) of collagen fibril size in papillary and reticular dermis of control (CT) and hyaluronic acid-treated (HA) skin samples at 24 and 48 h. The asterisks (*) indicate a statistically significant difference between HA-treated samples and the relative CT.

Following HA treatment, fibril size significantly increased at 24 h in both papillary and reticular dermis in comparison to control skin (both *p* < 0.001) (Figure 5). At 48 h, fibril size in the papillary dermis of HA-treated samples was significantly smaller than in the control (*p* < 0.001) (Figure 5), whereas no significant difference was found in the reticular dermis (*p* = 0.07). Notably, no statistically significant difference was found in both papillary and reticular dermis between values of HA-treated samples at 24 and 48 h (*p* = 0.84 and *p* = 0.06, respectively).

The interfibrillar distance was significantly larger at 24 h vs. 48 h in both the papillary and reticular dermis of control samples (*p* = 0.008 and *p* < 0.001, respectively). The values were statistically similar between the papillary and reticular dermis at both 24 h (*p* = 0.09) and 48 h (*p* = 0.53).

**Figure 6:**
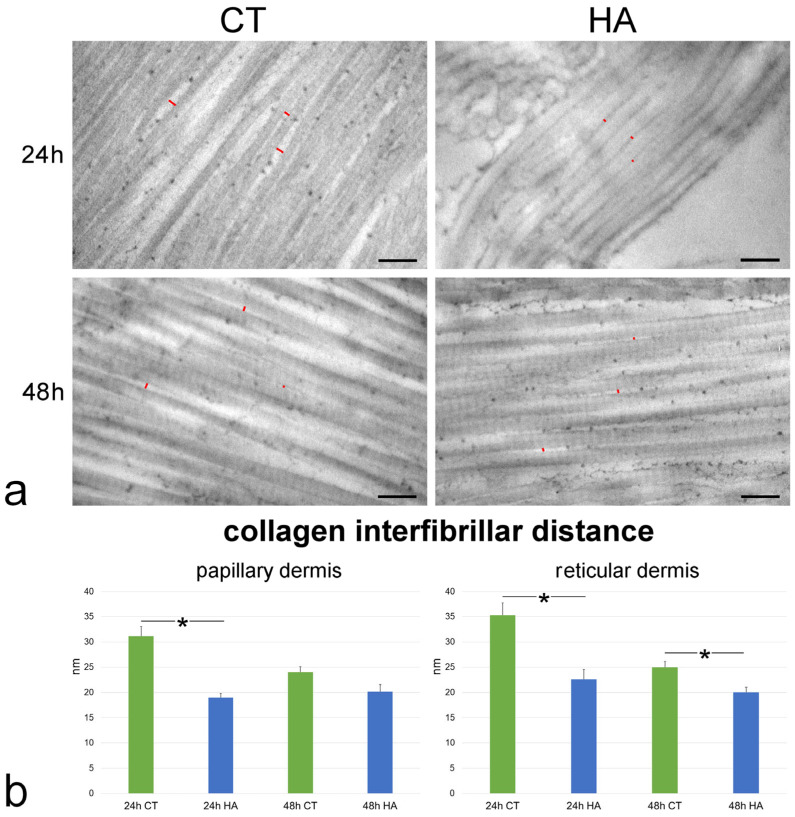
(**a**) TEM images showing collagen fibrils in the reticular dermis of control (CT) and hyaluronic acid (HA)-treated skin. Red lines indicate some interfibrillar distances. Bars, 200 nm. (**b**) Mean ± SE values (*n* = 40) of collagen interfibrillar distance in papillary and reticular dermis of control (CT) and hyaluronic acid-treated (HA) skin samples at 24 and 48 h. The asterisks (*) indicate a statistically significant difference between HA-treated samples and the relative CT.

HA treatment induced a significant decrease of the interfibrillar distance in comparison to control samples in papillary dermis at 24 h (*p* < 0.001) as well as in reticular dermis at both 24 and 48 h (both *p* < 0.001) (Figure 6). No significant difference was found between HA-treated and control samples in papillary dermis at 48 h (*p* = 0.06). Moreover, no statistically significant difference was found in both papillary and reticular dermis between values of HA-treated samples at 24 and 48 h (*p* = 0.30 and *p* = 0.07, respectively).

**Figure 7:**
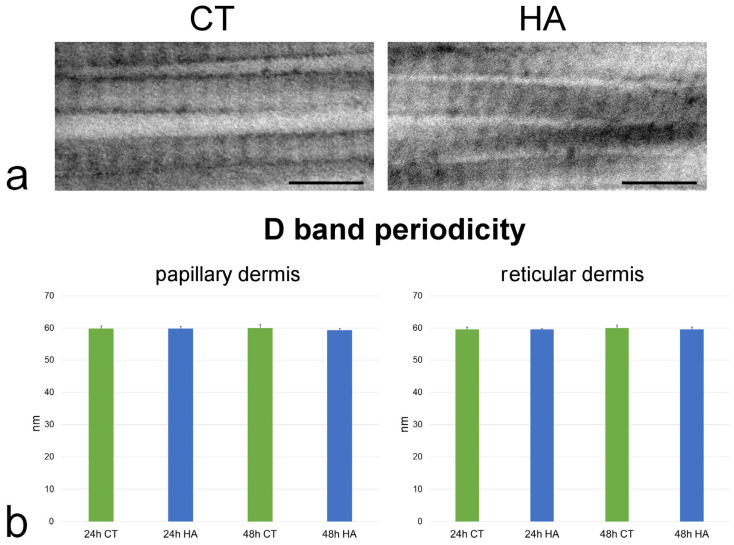
(**a**) TEM images showing high magnification details of collagen fibrils from reticular dermis of control (CT) and hyaluronic acid (HA)-treated skin after 48 h incubation. Bars, 200 nm. (**b**) Mean ± SE values (*n* = 100) of D band length in papillary and reticular dermis of control (CT) and hyaluronic acid-treated (HA) skin samples at 24 and 48 h. No statistically significant difference was found.

Collagen fibrils showed the typical D band periodicity and were associated in bundles with minimal interfibrillar distances in both papillary (Figure 6) and reticular dermis (Figure 6). Collagen fibril D band periodicity showed similar values in all samples, irrespective of dermis layer, timepoint, or treatment (*p* > 0.05 for all comparisons) (Figure 7).

## 3. Discussion

In this study we investigated the effect of in vitro transdermal administration of HA on the organization of dermal collagen in healthy human skin explants. The reliability of in vitro experimental models for preclinical studies is of primary importance; however, to date there are no models that can fully reproduce the structural complexity (i.e., the presence of all the cell types, the cell-to-cell and cell-to-matrix interactions, the appendages, and vessels) [20] and the neuro-immuno-endocrine signals transduction pathways [21] of human skin. Therefore, the use of human skin explants still is the best approach, although the restricted post-excision life span is a critical limiting factor [22]. To overcome this limitation, we have set up an innovative fluid dynamic system able to ensure a culture environment similar to the physiological one by an increased oxygen/nutrient supply, fast catabolite removal, and steady levels of temperature, humidity, O_2_, and CO_2_, which are all crucial factors to guarantee tissue survival in vitro. Under these conditions, the preservation of explanted organs and tissues proved to be prolonged [18,23], thus making them suitable for functional studies aimed at investigating, e.g., the permeation with drugs or nanoparticulates [24,25,26] or the inflammatory response [19]. In the bioreactor, the skin structural organization proved to be preserved, and in the present study, human skin explants were maintained in vitro for relatively long times (up to 48 h), allowing us to investigate the structural organization of the dermal collagen after HA transdermal administration by combining different microscopy techniques (LM, SEM, and TEM) [18].

Histological observations at LM showed similar structural features of the overlying epidermis and the underlying dermis in all skin explants [27]. In addition, SEM and TEM allowed observing the ultrastructural arrangement of the collagen fibril bundles, which were typically oriented in different directions in both papillary and reticular dermis, thus providing resistance to deformations. It is known that the mechanical properties of collagen-based tissues are strictly related to the hierarchical fibrillar structure of tropocollagen molecules capable of self-assembly due to their specific amino acid sequences [28]. In the skin, fibrils are arranged in a complex and interwoven network, allowing the tissue to withstand variously oriented forces. The way collagen fibrils are bundled affects the skin’ mechanical properties: in particular, densely packed, parallel bundles provide greater stiffness and strength, while looser, more disorganized bundles allow for greater flexibility and deformation [29].

Notably, quantitative assessment of the amorphous ECM component demonstrated higher values in the reticular vs. the papillary dermis in all samples. This confirmed the morphological observations of the lower compactness of the fibrillar arrangement in the reticular than in the papillary dermis [30]. The size of the collagen bundles was similar at 24 and 48 h in both the papillary and reticular dermis of control samples, and in all samples the collagen bundles resulted in being larger in the reticular than in the papillary dermis, as previously reported [30,31]. Notably, the typical D band periodicity of collagen fibrils due to the parallel and staggered arrangement of the assembled collagen monomers [32] was similar in all control samples, suggesting that no molecular alteration of the papillary and reticular dermis occurred during the permanence in the bioreactor. It is worth noting that the mean values of band length obtained in the present study are lower than 67 nm due to the well-known effect of tissue shrinkage caused by chemical fixation and are consistent with values reported in the literature [33].

Taken together, these findings confirm and extend the already proven reliability of our bioreactor in maintaining histological skin integrity by mimicking physiological conditions [18,19].

The application of the HA formulation to the healthy skin surface induces some structural modifications in the dermis. In fact, after 24 h treatment with HA, the reticular dermis showed a looser arrangement compared to the control samples, as clearly shown by LM and SEM images; these observations were supported by the morphometric analysis demonstrating a statistically significant increase in the amorphous ECM component. On the contrary, no change was found in the papillary dermis. The modifications observed in the reticular dermis may account for the hydrating effect of HA, which may be more evident in the deep dermis layer due to its loose arrangement than in the more compact papillary dermis. Several investigations have demonstrated that HA administration induces an increase in the ECM non-collagen components. First, HA is one of the ECM components whose content increases after external administration; as HA is highly hydrophilic, it attracts and holds water, improving the hydration of ECM and consequently increasing its volume [11]. By creating a hydrated and favorable environment, HA can also indirectly promote the synthesis of other ECM components such as proteoglycan and can modulate fibroblasts’ activity, increasing the synthesis of various ECM molecules [11,34,35]. All these effects likely contribute to the increase of the amorphous component of ECM observed in the reticular dermis in our study. However, after 48 h, the amorphous component in the reticular dermis was statistically similar to the controls, suggesting a temporary HA effect. The decrease of the amorphous ECM component in the papillary dermis of control samples after 48 h in comparison to 24 h may be accounted for by an occurring dehydration after a long incubation time, which may induce tissue shrinkage; this phenomenon is probably prevented in the reticular dermis by the abundant amorphous ECM component rich in hydrated molecules and/or by the direct contact of this dermis layer with the flowing medium in our system.

The reduction in thickness of collagen bundles observed in the reticular dermis after 24 h treatment with HA is likely related to the significant increase of amorphous ECM component occurring in these samples. In fact, no modification of collagen bundle thickness between control and HA-treated samples was observed in papillary dermis at 24 and 48 h or in reticular dermis at 48 h, where the amorphous ECM component did not undergo significant changes after HA treatment. Probably, an HA-induced increase of the amorphous matrix took place, causing a physical separation between collagen bundles, thus compressing the fibrillar component, although this effect was transitory and the bundles restored their original size [36,37]. These results obtained also highlight also the involvement of HA in the aging process. In fact, as noticed by Lavker and colleagues in a study focused on the comparison between human skin from old and young donors using light, transmission, and scanning electron microscopy, one of the most peculiar changes noted in aged skin is the “apparent increase in density of the collagen network” due to the aging-linked decrease of the amorphous ECM matter placed between the individual collagen bundles [38,39]. As to the anti-aging effect of HA, our findings demonstrate that, in samples treated with HA, the ECM amorphous component increased, giving the skin a morphological appearance typical of young skin. Moreover, Lavker’s group pointed out that the packing of the fibrils in aged skin is not as tight as in the young, and this observation is consistent with our ultrastructural analysis, which revealed a smaller collagen interfibrillar distance in samples treated with HA, once again giving the skin a youthful appearance.

In the papillary dermis at 48 h post-treatment, collagen bundles became more linear in control samples; on the contrary, HA was able to maintain a more tortuous pattern, while in the reticular dermis, HA increased collagen bundle tortuosity at 24 h: this demonstrates that the HA treatment may also influence the tortuosity of the collagen bundles. It has been reported that dermal collagen fibers are characterized by a tortuous trend that is progressively lost with increasing age [40], in parallel with a decrease in the amorphous ECM [41] and a loss of hydration due to proteoglycan modifications [42]. Interestingly, it has been demonstrated that the interaction between water molecules and proteoglycans may influence the packing of collagen fibrils and the orientation of collagen bundles, influenced also by the cellular behavior that is in turn dependent on the influence of HA [43]. In this view, the capability of HA to maintain collagen bundle tortuosity would likely favor tissue hydration and space filling, thus contributing to the anti-aging effect of this molecule [44].

The high resolution of TEM allowed us to observe some changes induced by HA treatment also at the level of individual collagen fibrils. Fibril size significantly increased after 24 h HA treatment in both papillary and reticular dermis, but the D band periodicity did not change, thus excluding alterations in collagen monomer assembly. It is known that, when collagen fibrils are hydrated, water molecules penetrate the intrafibrillar space, and this influx of water causes fibril swelling, leading to an increase in their diameter [45]. HA attracts and holds water, enhancing the collagen swelling effect, while HA depletion induces reduction of the fibril size [46]. The parallel decrease in interfibrillar distance observed in both papillary and reticular dermis after 24 h HA treatment is likely a consequence of the enlargement of fibril size. Accordingly, some studies showed that hydration modulates protein interactions within the collagen fibrils, thus influencing their separation [47,48].

At 48 h, in both the papillary and reticular dermis of control samples, the collagen fibril size increased and the interfibrillar distance decreased in comparison to 24 h. Conversely, HA-treated samples maintained the fibril size and interfibrillar distance values observed at 24 h. It is known that HA plays a significant role not only in determining collagen fibril size but also in stabilizing fibril arrangement within the ECM; in fact, the HA hydration effect prevents collagen fibrils from disorganization and maintains the proper interfibrillar spacing [49].

Despite the promising results obtained in this study, it is worth to note that these findings have been obtained on a single skin explant from a 51-year-old woman. It is well known that multiple factors may influence the ECM response to HA. For instance, skin aging may modify the type and amount of ECM molecular components, thus compromising its structure and its water-binding capacity [50]. Moreover, genetic factors responsible for the expression and function of ECM proteins and enzymes involved in ECM remodeling may induce skin variability from donor to donor [51]. Finally, differences in skin structure and aging pattern are present between different ethnicities, possibly influencing ECM response to exogenous HA [52].

## 4. Materials and Methods

### 4.1. Samples Preparation

**Figure 8:**
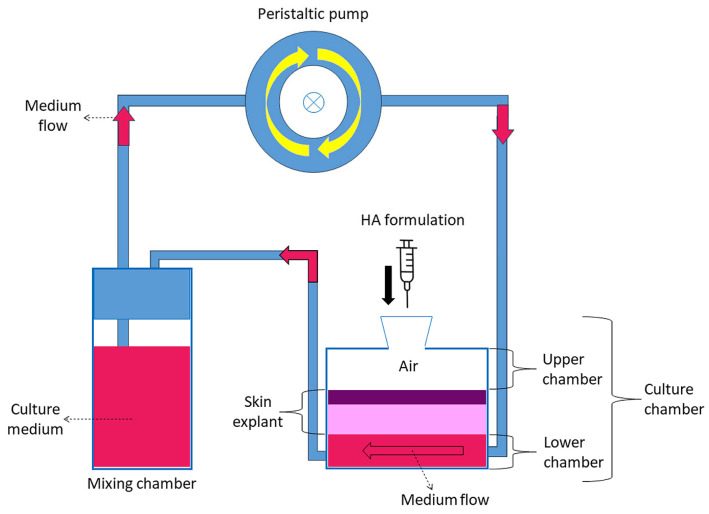
Schematic representation of the bioreactor.

Healthy human skin samples were obtained as waste material from reduction breast surgery performed on a 51-year-old female patient, in compliance with the Declaration of Helsinki of the World Medical Association and signed informed consent. During transportation from the operating room to the research laboratory, the skin explants were kept at 4 °C. Upon arrival, the skin samples were quickly washed at room temperature in a physiological solution (0.9% *w/v* NaCl) and then in pre-warmed culture medium at 37 °C (DMEM medium, supplemented with 10% *v/v* fetal bovine serum, 1% *v/v* penicillin-streptomycin, 1% *v/v* L-glutamine, and 0.5% *w/v* amphotericin B). All reagents were purchased from ThermoFisher Scientific Inc. (Waltham, MA, USA). The subcutaneous fatty tissue was removed to expose the dermis. Circular samples with a diameter of 1.5 cm were cut and mounted in the bioreactor chambers (IV-Tech, Massarosa, Lucca, Italy) (Figure 8), with the stratum corneum facing upward (in contact with air) and the dermis facing downward (in contact with the circulating culture medium). This setting mimics the physiological condition of human skin with the epidermis exposed to the air and supplied with nutrients by the underlying dermis by diffusion.

Control and HA-treated samples were analyzed at 24 h and 48 h time points in duplicate. In detail, two circular samples of skin were used for each experimental condition and time point. The two samples were placed in distinct culture chambers of the bioreactor and processed simultaneously; moreover, both samples were used for morphological and morphometrical analyses.

A total of 9 mL of pre-warmed medium was placed in the mixing chamber connected in parallel to a peristaltic pump, ensuring the flow of culture medium in contact with the dermis; the flow rate was set at 500 µL/min since it was found to guarantee the best preservation of explanted skin [18]. The bioreactor was kept in an incubator at 37 °C with a humidified atmosphere of 5% CO_2_. The culture medium was replaced every 24 h. 300 µL of “Skin-F 24” (Italfarmacia S.r.l., Rome, Italy) namely, a hydrogel composed of highly purified 24 mg/mL cross-linked sodium hyaluronate-based of non-animal origin, produced by bacterial fermentation. The cross-linked sodium hyaluronate in sterile buffered water, with amino acids glycine and proline added, was applied to the stratum corneum of the skin samples placed in the bioreactor immediately after activating the dynamic flow system. Skin-F 24 is designed for intradermal injection but, in our protocol, it was placed on the surface of the skin and left on for 24 h or 48 h to simulate transdermal administration. As a control, some skin samples were maintained in the bioreactor but without HA treatment. Control and treated samples were analyzed at 24 h and 48 h time points in duplicate (i.e., skin samples were assembled in parallel in two different bioreactors and subjected to the same experimental conditions). All samples were processed for LM, SEM, and TEM.

### 4.2. Light Microscopy

For LM analysis, skin samples were fixed in 4% *w/v* paraformaldehyde and subsequently embedded in paraffin wax [18]. For histological evaluation, 7-µm-thick cross-sections, including both the epidermal surface and the whole dermis, were stained using hematoxylin and eosin solution (BioOptica, Milan, Italy) in the same run, in order to obtain homogenous staining and allow reliable signal intensity evaluation. The samples were examined with a light microscope equipped with a 20× objective and interfaced with a digital camera (Olympus BX63-CBH with Retiga 2000R camera, Center Valley, PA, USA). In order to detect changes in the ECM organization, 40 images per condition (20 per duplicate) were analyzed with a MATLAB code 2023a developed to measure the number of unstained pixels (considered as the amorphous component of ECM) over the number of eosin-stained pixels (considered as the fibrillar component of ECM, mainly made of collagen fibers). Specifically, the pixels with intensity over a threshold defined by an expert author not aware of the experimental group of the images were considered as unstained pixels. The results were expressed as the percentage of amorphous ECM. For the morphometric measures, the dermal compartment only was considered; in particular, the papillary dermis (occurring just beneath the epidermis) and the reticular dermis (forming the deeper dermal region) were separately evaluated.

### 4.3. Scanning Electron Microscopy

Skin samples for SEM analysis were fixed with 2% *w/v* paraformaldehyde and 2.5% *v/v* glutaraldehyde, and post-fixed with 1% *v/v* OsO_4_ and 1.5% *v/v* potassium ferrocyanide (all reagents from Electron Microscopy Sciences, Hatfield, PA, USA). Subsequently, the specimens were dehydrated using a graded series of acetones, processed using a critical point dryer (CPD 030; BAL-TEC AG, Balzers, Liechtenstein), mounted on aluminum stubs with sticky carbon, and coated with gold (MED 010; BAL-TEC AG). Observations were carried out using an XL 30 ESEM (FEI, now part of Thermo Fisher Scientific Inc.) scanning electron microscope. Images of cross sections of the skin were acquired, in order to distinguish papillary dermis and reticular dermis.

### 4.4. Transmission Electron Microscopy

For TEM, samples were fixed with 2% *w/v* paraformaldehyde and 2.5% *v/v* glutaraldehyde, post-fixed with 1% *v/v* OsO_4_ and 1.5% *v/v* potassium ferrocyanide, and finally embedded in Epon-Araldite resin (all reagents from Electron Microscopy Sciences) [18]. Ultrathin sections (70–80 nm in thickness) were stained with lead citrate and observed using a Philips Morgagni transmission electron microscope (FEI) operating at 80 kV and equipped with a Megaview II digital camera. Images of longitudinally sectioned collagen bundles were used for morphometric evaluation of collagen bundle thickness, collagen bundle linearity (an index expressed as the ratio between the real length of the bundle profile and the corresponding linear length), fibril size, interfibrillar distance [53,54], and D band length (periodicity). For each variable, one measure per bundle or fibril was made. TEM images were taken at 8900× for bundle thickness and linearity, and at 36,000× for the other variables in both papillary and reticular dermis per each experimental condition. For collagen bundle thickness, collagen bundle linearity, fibril size, and interfibrillar distance, 40 measurements per condition (20 per duplicate) were made, while for D band length, 100 measurements per condition (50 per duplicate) were made.

### 4.5. Statistics

Quantitative values for individual variables were pooled according to the experimental condition (control and HA-treated samples), timepoint (24 h and 48 h) and dermis layer (papillary and reticular dermis), and presented as mean ± standard error of the mean. For D band periodicity, statistical paired comparisons were carried out with the *t*-test. Since the other variables showed non-normal distribution, paired comparisons were performed with the non-parametric Mann–Whitney test. Statistical significance was set at a *p* value < 0.05.

## 5. Conclusions

In this study an innovative fluid dynamic system was used to investigate the effects of a cross-linked macrostructural HA formulation on dermal collagen of healthy human skin explants. The good preservation of skin explants provided by the bioreactor allowed applying refined high-resolution microscopy techniques to analyze in situ the HA-induced modifications on the ECM collagen fibrils up to 48 h from the application on the skin surface.

Although our findings need further consideration about skin variability (e.g., age, genetic factors, ethnicities), the present study demonstrates that the HA formulation that is commercially proposed for subcutaneous injection may act on dermal ECM also when applied transcutaneously: in our skin model ex vivo, we found that the ECM hydration was improved and the organization of the collagen fibrils modified, and this is consistent with the volumizing and anti-aging effect of HA.

Non-invasive skin rejuvenation strategies may therefore be foreseen even for products that were originally intended for more invasive administration routes. Further studies are in progress on long term effects on skin collagen of single HA administration as well as of multiple HA treatments.

## Data Availability

The raw data supporting the conclusions of this article will be made available by the authors on request.
